# Review: The role of HMGB1 in spinal cord injury

**DOI:** 10.3389/fimmu.2022.1094925

**Published:** 2023-01-12

**Authors:** Yizhang Mo, Kebing Chen

**Affiliations:** Department of Spine Surgery, The Sixth Affiliated Hospital of Sun Yat-sen University, Guangzhou, China

**Keywords:** HMGB1, spinal cord injury, inflammation, neuron, mechanism

## Abstract

High mobility group box 1 (HMGB1) has dual functions as a nonhistone nucleoprotein and an extracellular inflammatory cytokine. In the resting state, HMGB1 is mainly located in the nucleus and regulates key nuclear activities. After spinal cord injury, HMGB1 is rapidly expressed by neurons, microglia and ependymal cells, and it is either actively or passively released into the extracellular matrix and blood circulation; furthermore, it also participates in the pathophysiological process of spinal cord injury. HMGB1 can regulate the activation of M1 microglia, exacerbate the inflammatory response, and regulate the expression of inflammatory factors through Rage and TLR2/4, resulting in neuronal death. However, some studies have shown that HMGB1 is beneficial for the survival, regeneration and differentiation of neurons and that it promotes the recovery of motor function. This article reviews the specific timing of secretion and translocation, the release mechanism and the role of HMGB1 in spinal cord injury. Furthermore, the role and mechanism of HMGB1 in spinal cord injury and, the challenges that still need to be addressed are identified, and this work will provide a basis for future studies.

## 1 Introduction

High mobility group box 1 (HMGB1), also known as amphotericin or HMG1, is a nonhistone chromatin binding protein first discovered in the 1960s. HMGB1 shows high electrophoretic mobility when run on polyacrylamide gels, hence its name (1, 2). HMGB1 is highly conserved in evolution, and the HMGB1 in rodents shares 99% homology with that in humans (3–5). HMGB1 is also expressed partially in the cytoplasm because it shuttles back and forth from the nucleus (6). HMGB1 has the dual functions of a nonhistone nucleoprotein and an extracellular inflammatory cytokine. HMGB1 binds extensively to DNA in the nucleus and participates in transcriptional regulation, DNA replication and repair, telomere maintenance and nucleosome assembly (7). Extracellular HMGB1 is passively released or actively secreted by necrotic tissue or stress cells. As a chemokine or cytokine, it binds to pattern recognition receptors (PRRs) to create a damage-associated molecular pattern (DAMP) (6–12). HMGB1 plays an important role in many diseases, including traumatic shock, fatty liver disease, septicaemia, autoimmune diseases, and cancer (8, 11, 13–15).

In recent years, the role of HMGB1 in spinal cord injury (SCI) has attracted a great deal of attention. SCI is characterized by sensory, motor and autonomic nerve dysfunction (16) mediated by complex and diverse pathophysiological processes, including neuroinflammation, neuronal death, glial scar formation and axonal regeneration (17–20). The concentration of HMGB1 in the injured area increases rapidly and lasts for a long time (21–31), and it not only aggravates injury by playing the role of an inflammatory cytokine, but it also promotes the recovery of the injured spinal cord.

This article reviews the research progress of studies investigating the expression and release of HMGB1 after SCI, its effect on the injured spinal cord and its potential therapeutic mechanism.

## 2 Main text

### 2.1 The structure of HMGB1 and the lower function of the resting state

The HMGB1 protein is a highly conserved nuclear protein that consists of 215 amino acids and has a molecular weight of approximately 30 kDa. Structurally, HMGB1 is divided into the following three functional regions: the A-box, the B-box and the acidic C-terminus. The A-box and B-box are composed of 80-90 amino acid residues with similar amino acid repeat sequences and nonspecific DNA-binding sites, and the B-box is the functional structural region that causes inflammation (32, 33); however, the A-box has a certain antagonistic effect on the B-box (34). The acidic C-terminus containing aspartic acid and glutamate is mainly involved in regulating the binding of HMGB1 to DNA and mediating gene transcription and chromosome unwinding (35). The B-box domain has two key binding sites, Toll-like receptor 4 (TLR4) and a receptor for advanced glycation end (RAGE) products, which regulate the release of proinflammatory cytokines (36, 37). HMGB1 has two nuclear localization sequences (NLSs); one is in the A-box, the other is between the B-box and the C-terminal tail, and the nuclear export signal (NES) is contained in the DNA-binding domain (38).

During the resting state of HMGB1, it is located mainly in the nucleus and it regulates key nuclear activities, including transcription, replication, DNA repair and nucleosome formation, all of which are important for maintaining steady-state cellular function (7, 38).

Collectively, these results show that HMGB1 is a highly conserved protein with proinflammatory and anti-inflammatory potential. In the resting state, HMGB1 is located mainly in the nucleus and regulates physiological processes there; however, there are no related studies on the structural and functional changes to HMGB1 in the nucleus after SCI.

### 2.2 Expression of HMGB1 after SCI

After SCI, HMGB1 is expressed and released by neurons, microglia/macrophages, and ependymal cells (21–23). After injury, the expression of HMGB1 is rapidly upregulated and released into the extracellular matrix and circulating blood, which lasts for a long period of time. In a rat experiment, the expression of HMGB1 mRNA and HMGB1 protein was found to be upregulated 2-6 hours after SCI, the peak level was reached 1-3 days after injury, and this enhanced level lasted for 28 days after SCI (21, 23, 39, 40); however, the number of HMGB1 positive cells in the spinal cord of injured rats was the highest 48 hours after injury (41). In addition, Fan et al. found that the concentration of HMGB1 in serum increased significantly 3 days after SCI (42). In a model of SCI using neurons *in vitro*, we found that after injury, the concentration of HMGB1 in the culture medium immediately increased to 5 ng and 17 ng/ml at 6 and 12 hours, respectively, and reached 19 ng/ml HMGB1 at 24 hours (27). Interestingly, in addition to the increased expression of HMGB1 in the acute and subacute phases after SCI, the level of HMGB1 also increased significantly in the chronic phase. Papatheodorou et al. found that HMBG1 levels also increased significantly in patients 5 or more years after SCI (43). However, the findings of Fang et al. in zebrafish experiments suggested different expression patterns of HMGB1, and these results contradict the continuous increase in HMGB1 found by most studies. The level of HMGB1 mRNA increased twofold at 4 hours after SCI, decreased 12 hours and 11 days after injury, and increased again 21 days after injury (22).

Collectively, these results show that the expression of HMGB1 increases rapidly after SCI and lasts for a long time, even throughout the acute, subacute, and chronic stages, implying that HMGB1 may affect the severity of SCI and the process of recovery. In addition, the expression of HMGB1 was still upregulated in the chronic phase after injury, and its specific mechanism and effect still need to be explored. Moreover, the specific mechanism of the new model of HMGB1 expression remains to be further validated. Reguarding the role of HMGB1, we speculate that the first increase in HMGB1 expression is beneficial to protect injured neurons, then the decrease in HMGB1 expression is beneficial to reduce inflammation and further reduce neuronal death, and then the continuous increase in expression helps promote nerve regeneration and recovery of the injured spinal cord.

### 2.3 Translocation time of HMGB1 after SCI

After SCI, the expression of HMGB1 increases rapidly, translocates from the nucleus to the cytoplasm; furthermore, it may be released into the extracellular matrix through cytoplasmic vacuoles. Two hours after SCI, the nuclear level of HMGB1 increased significantly, and HMGB1 in the nucleus was gradually released into the cytoplasm. Six hours after injury, the cytoplasmic level of HMGB1 increased significantly. HMGB1 is located mainly in the nucleus of neurons in the early stages after SCI, and then it translocates to the cytoplasm a few hours later (21, 27, 44). Interestingly, in a zebrafish SCI model, Fang et al. found that HMGB1 was in the cytoplasm of motoneurons 4 hours after injury; however, 12 hours after injury, the cytoplasmic HMGB1 level in motoneurons decreased. On the 21st day of SCI, the level of HMGB1 in the cytoplasm decreased, but HMGB1 was again detected in the nucleus. HMGB1 exists in the motoneuron nucleus and it translocates to the cytoplasm after injury; furthermore, its expression is downregulated in motoneurons (22). The most significant change in HMGB1 translocation after SCI in mice was in the macrophages at the lesion centre and near the lesion boundary. HMGB1 could not even be detected in the nucleus, while it was still found in small cytoplasmic vacuoles, implying that HMGB1 may be packaged in the cell to be secreted into the extracellular space (23).

Taken together, these results indicate that HMGB1 increased in the nucleus and was released from the nucleus to the cytoplasm and extracellular matrix; however, the specific time and node of translocation have not been clarified.

### 2.4 Possible mechanism of HMGB1 release after SCI

The mechanism of secretion and release of HMGB1 in SCI has not been specifically explained, but it is generally thought that HMGB1 mainly comes from the secretion of inflammatory cells and the release of dead neurons (21–23, 45, 46).

In SCI, HMGB1 may carry out nuclear and cytoplasmic transport through acetylation, phosphorylation and methylation of the nuclear localization sites (NLS). In a study of *T. thermophila* CU 427 and CU 428, two NLSs were found to control the nuclear localization of HMGB1 in the steady state (47). Posttranslational modifications of the NLS site, including acetylation, phosphorylation and methylation, regulate the ability of HMGB1 to be transported to the cytoplasm during cellular stress (48–51). In a study of mouse fibroblasts, HeLa cells and Saos-2 cells, it was found that excessive acetylation of lysine at the NLS site was essential for the translocation of HMGB1 from the nucleus to the cytoplasm in monocytes stimulated by LPS, TNF or IL-1. Inhibiting the peracetylation of the NLS, can inhibit the translocation of HMGB1 from the nucleus to the cytoplasm and block the translocation of HMGB1 from the cytoplasm to the nucleus (2, 48, 52). In addition, the NLS site of HMGB1 was found to be phosphorylated and translocated to the cytoplasm of mouse macrophages stimulated by TNF (53). In neutrophils, the methylation of the lysine site of HMGB1 NLS was found to weaken the DNA-binding activity of HMGB1, resulting in the passive diffusion of HMGB1 out of the nucleus (49).

In SCI, the mechanism of extracellular secretion of HMGB1 is related to inflammation. Inflammation can induce many kinds of cells to secrete HMGB1 (54, 55); however, HMGB1 cannot be actively secreted through the conventional endoplasmic reticulum-Golgi secretion pathway utilized by most soluble secretory proteins (56, 57). At present, scholars have proposed two forms of active release of HMGB1 (58). One is stimulation and activation of the target cells, causing HMGB1 to be secreted into the outer space of the cells (48, 59). The second is packaging HMGB1 into intracellular vesicles, and then releasing HMGB1 outside the cell after the vesicles fuse with the cell membrane (60, 61). The latter is consistent with HMGB1 being in cytoplasmic vacuoles in macrophages after SCI (23).

HMGB1 translocation and release during aseptic inflammation can be regulated by calcium-mediated signal transduction (50, 62, 63). Phosphorylation and release of HMGB1 are regulated by activation of calcium-mediated protein kinases, especially calmodulin-dependent protein kinases (CaMKKs) (64–67). Calcium signal inhibitors inhibit HMGB1 secretion and protect animals in various disease models (50, 68). In addition, heat shock protein family A (Hsp70) member 1A (HSPA 1A, also known as HSP72) can block HMGB1 secretion in macrophages by inhibiting the interaction between HMGB1 and XPO1 (69). In different inflammation and injury models, peroxisome proliferator activated receptor (PPAR) binds to specific ligands and activates the transcription of PPAR target genes. The secretion of HMGB1 in activated macrophages is negatively regulated by PPAR (70). In contrast, JAK-regulated STAT1 and STAT3 activation plays an active role in the expression, modification and/or release of HMGB1 (71–75), while extracellular HMGB1 can trigger the activation of the STAT1 and STAT3 pathways (74–78). In addition, MAPK family members and inflammatory bodies promote the release of HMGB1 in different inflammatory and injury models (79–82). Deficiency of complement 5a receptor 2 (C5aR2) limits the activation of NLRP3 inflammatory bodies and the release of HMGB1 *in vitro* (83).

Expression of the inflammatory cytokines TNF and NF-κB were upregulated after SCI. TNF knockout or a TNF neutralization antibody directly inhibited TNF and partially inhibited HMGB1 release induced by IFN-γ and LPS in macrophages, indicating that the secretion of HMGB1 is partially mediated by a TNF-dependent mechanism (84). In addition, the NF-κB pathway is involved in the release of HMGB1, and the inhibition of the classical NF-κB pathway limits the secretion of HMGB1 in activated immune cells; however, the target gene of NF-κB that causes the secretion of HMGB1 is still unknown (85–87). Furthermore, the involvement of TNF (the classical NF-κB target gene) in the NF-κB dependent release of HMGB1 cannot be ruled out.

After SCI, HMGB1 can be passively released after various types of cell death in response to various stimuli or injuries. In addition, the release of many intracellular substances (cathepsin, antioxidant enzymes, DNase, caspases) after cell death can also promote the secretion of HMGB1 by inflammatory cells (81, 88–100).

**Figure 1:**
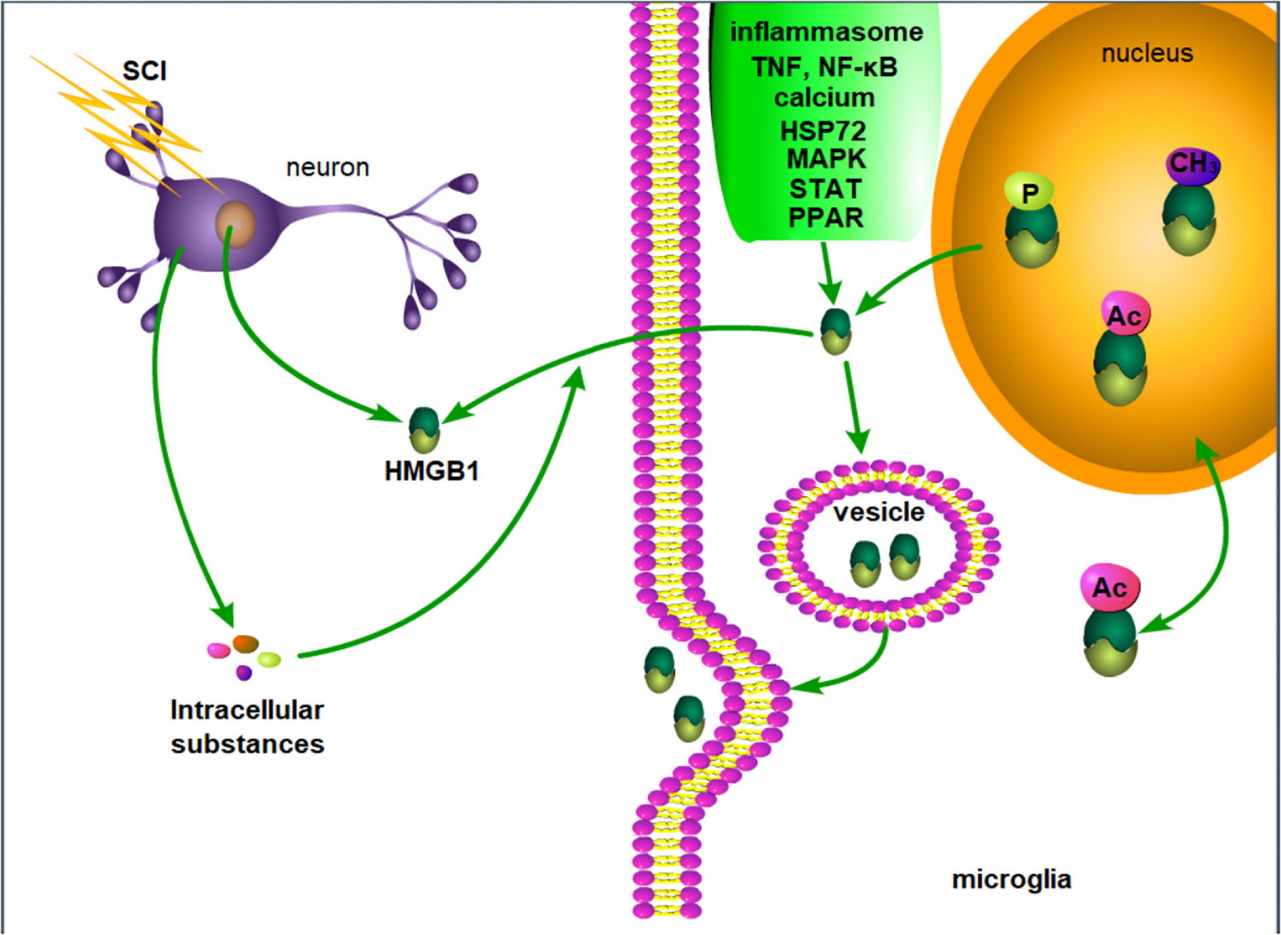
Possible mechanism of HMGB1 release after SCI. After SCI, injured neurons passively release HMGB1, and the intracellular substances released by necrotic neurons and inflammation lead to the active release of HMGB1 by microglia. P, phosphorylation. Ac, acetylation. CH3, methylation.

In summary, although the secretion and release mechanism of HMGB1 in SCI has not been specifically described, the release mechanism of HMGB1 described in other fields if of great value. In SCI, HMGB1 may be actively released by inflammatory cells or passively released by necrotic neurons, and intracellular substances released by necrotic cells may further induce the active secretion of HMGB1 (**Figure 1**).

### 2.5 HMGB1 may regulate cell migration after SCI

Although the role of that HMGB1 plays in inducing cell migration in SCI has not been reported, in other diseases, HMGB1 has been found to induce cell migration by activating RAGE or activating the CXCR4 receptor through a heterologous complex with CXCL12. Many studies have shown that HMGB1, as a potential host cell-derived chemokine, promotes the migration of nerve processes and many cell types. These cells include smooth muscle cells, myoblasts, tumour cells, hepatic stellate cells, stem cells, endothelial cells, keratinocytes, monocytes, dendritic cells and neutrophils (101–117).

RAGE is necessary for HMGB1-mediated cell migration. HMGB1 triggers RAGE to induce the transcription of chemokine genes such as CCL3, CCL4 and CXCL12, while HMGB1-induced migration can quickly be blocked by anti-RAGE or anti-HMGB1 neutralizing antibodies, confirming the importance of RAGE in the process of migration (102, 118–121). Recent studies have shown that the triggering of RAGE by HMGB1 induces the transcription of chemokine genes such as CCL3, CCL4 and CXCL12, and subsequently it participates in cell migration (118, 119, 121). In addition, HMGB1 can induce cell migration by forming heterocomplexes with CXCL12 is mediated by CXCR4 receptors, and the HMGB1-CXCL12 complex is more effective in inducing monocyte migration than CXCL12 alone (109, 122–124). By activating CXCR4 receptors, HMGB1-CXCL12 complexes recruit leukocytes from the circulation, and then induce leukocytes to activate and secrete cytokines and chemokines, thus promoting inflammation (125, 126). Interestingly, HMGB1 not only stimulates but also inhibits migration in some cells. For example, exogenous HMGB1 selectively inhibits VEGF-induced cell migration in pulmonary artery endothelial cells (HPAECs), but does not inhibit human umbilical vein endothelial cells (HUVECs). In addition, the IRF3-dependent TLR4 pathway is necessary for HMGB1-mediated inhibition of migration in HPAECs (115).

Collectively, these data show that HMGB1 can promote the migration of a variety of cells, including inflammatory cells and stem cells. Although the chemotaxis of HMGB1 has not been studied in SCI, it remains possible that HMGB1 could induce inflammatory cell migration to aggravate the inflammatory response or promote injury recovery by inducing stem cell migration following SCI.

### 2.6 HMGB1 induces an inflammatory response after SCI

HMGB1 aggravates the inflammatory response after SCI. We found that after SCI, HMGB1 and its receptors were significantly colocalized in the white matter of the injured rat spinal cord (21). After HMGB1 injection, the focus of activated microglia was obviously in the ventral horn of the spinal cord (23), but the lesion area of anti-HMGB1 mAb-treated mice was significantly smaller than the lesion area of untreated mice (127–129). HMGB1 is thought to aggravate SCI. To further investigate its specific mechanism, Nakajo et al. found that anti-HMGB1 mAb treatment could protect the BSCB from damage caused by SCI, reduce the level of AQP4 protein in the spinal cord, and inhibit the swelling of the injured spinal cord. Anti-HMGB1 mAb inhibits inflammation after SCI in the early acute phase and it can prevent BSCB destruction directly or indirectly by inhibiting the expression of inflammatory cytokines and MMP in SCI model mice. Anti-HMGB1 mAb can relieve SCI, oedema and demyelination, thus promoting the recovery of the spinal cord (27, 128).

After SCI, HMGB1 induces inflammation mainly by activating microglia rather than astrocytes. Colocalization of microglial activation and obvious neuronal loss occurs in the spinal cord of rats with HMGB1 injection (23). HMGB1 has been reported to induce inflammation by activating microglia/macrophages (21, 44). In addition, further studies found that HMGB1 contributes to the development of the neurotoxic inflammatory macrophages (M1) phenotype after SCI (23). The mRNA levels of TNF-α, iNOS and CD86 increased significantly in microglia treated with HMGB1, which provided direct evidence for the activation of microglia to the M1 phenotype by HMGB1. HMGB1 or RAGE inhibition can inhibit the activation of macrophages/microglia to the M1 phenotype and promote the activation of macrophages/microglia to the M2 phenotype after SCI (27, 42, 130). Moreover, recombinant HMGB1 promoted the migration of BV2 microglia, while the anti-HMGB1 polyclonal antibody weakened the migration of BV2 microglia. The secretion of HMGB1 after SCI has been reported to recruit microglia to participate in the inflammatory response (27); however, HMGB1 failed to activate the classical inflammatory signalling pathway of primary astrocytes, indicating that astrocytes may not be induced by HMGB1.

After SCI, HMGB1 regulates the expression of inflammatory factors and the inflammatory response through RAGE and TLR2/4. HMGB1,TNF-α and RAGE are expressed in the same apoptotic neurons after SCI (41). There was a significant correlation between the levels of HMGB1 and NF-κB and the expression of TLR4 and NF-κB protein after SCI (131). HMGB1, TNF-α and RAGE are expressed in the same apoptotic neurons, and the temporal expression patterns of RAGE and HMGB-1 are similar (21, 41). The expression of RAGE, TNF-α, NF-κB IFN-γ, IL-1α, IL-6 and IL-17 increased in microglia treated with HMGB1, while inhibition of HMGB1 decreased the levels of RAGE, TNF-α, NF-κB IFN-γ, IL-1α, IL-6 and IL-17 (27, 42). HMGB1 induced inflammation by activating TLR2/4 or RAGE, activating its downstream pathway and upregulating the levels of TNF-α and NF-κB (28, 132–136). Interestingly, Wang et al. found that the administration of recombinant HMGB1 did not increase the levels of inflammatory cytokines TNF-α and IL-1β, while blocking RAGE reduced the induction of cytokines induced by LPS. It has been proposed that HMGB1/RAGE does not directly increase proinflammatory cytokines, and the effect of RAGE on cytokines may be related to pathogen-related interactions (137).

Collectively, these results shown that the expression of HMGB1 is upregulated after SCI, the inflammatory response is aggravated by regulating the activation of microglia to the M1 phenotype, and the expression of inflammatory factors is regulated by RAGE and TLR2/4. However, the studies of Wang et al. indicate different possibilities, and the specific mechanism remains to be further validated. In addition, these studies investigated the role of HMGB1 in only acute and subacute SCI. However, as the expression of HMGB1 is still upregulated in the chronic phase of SCI, its specific role needs to be further explored. Finally, extracellular HMGB1 can aggravate the inflammatory response after SCI, but the specific changes in nuclear HMGB1 after SCI are not clear, and its specific role needs to be understood.

### 2.7 Effect of HMGB1 on injured neurons after SCI

After SCI, HMGB1 promotes neuronal death by promoting nerve inflammation. After HMGB1 injection, focus of activated microglia and the area of neuronal loss were determined. Coculture of macrophages stimulated by HMGB1 with neurons showed a decrease in axonal growth (23). Inhibition of the HMGB1/TLR4/NF-κB signalling pathway can inhibit neuroinflammation and apoptosis in SCI, reduce the damage, oedema and demyelination, improve the survival rate of host neurons, and promote the recovery of the spinal cord (24, 27, 28, 127, 128, 138–140) Interestingly, Song et al. found that transplantation of HMGB1-preconditioned neural stem cells can promote neuronal survival after SCI in rats, promote the connection between relay neurons and motor neurons; furthermore, the newly formed neural circuits greatly improved motor recovery (25, 30). These findings are consistent with the findings of Wang et al. in cerebral ischemia-reperfusion injury. HMGB1 preconditioning can significantly reduce neurological deficits, infarct size, brain swelling, apoptosis and blood-brain barrier permeability in rats with cerebral ischemia-reperfusion injury (141). In addition, RAGE blockade was shown to not be conducive to neuronal survival after SCI (137), and consistent with the findings of Huttunen et al. HMGB1 can promote neuronal survival and axonal growth by activating RAGE and increasing the expression of the anti-apoptotic protein Bcl2 (142–145). HMGB1 is thought to be beneficial for the survival of injured spinal cord neurons.

In summary, numerous studies have shown that HMGB1 can exacerbate neuronal injury by aggravating neuroinflammation. Some studies have also suggested that HMGB1 is conducive to the survival and recovery of injured neurons. However, the specific mechanism by which HMGB1 protects injured neurons and promotes injury recovery is not clear, and this does not rule out the possibility that HMGB1 indirectly injures neurons by promoting the inflammatory response and protects neurons through direct action.

### 2.8 HMGB1 induces neuronal regeneration after SCI

After SCI, HMGB1 promotes the growth of neuronal axons and induces the differentiation of neural stem cells. Fang et al. found that HMGB1 increased significantly and the number of perivascular motoneurons increased significantly 6th days after SCI. After inhibition of HMGB1, the number of detected axons decreased significantly. HMGB1 is thought to participate in axonal regeneration and promote motor recovery (22). Further *in vitro* experiments showed that HMGB1, especially when close to the neuronal cell body, significantly increased neuronal axonal growth (23). In addition, HMGB1 can also promote neuronal differentiation. HMGB1 can induce the differentiation of neural stem cells through the ERK signalling pathway and the RAGE signalling pathway, while antagonism of these pathways can reduce the expression of marker molecules in mature neurons (25, 137). These results indicate that HMGB1 can induce the differentiation and maturation of neural stem cells through the ERK signalling pathway and the RAGE signalling pathway. Interestingly, Palumbo et al. found that HMGB1 can induce stem cells to migrate across the endothelial barrier *in vitro* and *in vivo* (104). Thus, the role of recruiting stem cells in the recovery of SCI remains to be explored.

Collectively, these results show that HMGB1 can promote the growth of neuronal axons and induce the differentiation of neural stem cells after SCI; although, the specific mechanism needs to be further explored. In addition, in the chronic stage of SCI, the upregulation of HMGB1 may be involved in regulating neuronal regeneration and promoting motor recovery.

### 2.9 HMGB1: A potential target for clinical treatment after SCI

After SCI, HMGB1 aggravates the damage by inducing inflammation, and after treatment with hyperbaric oxygen, shikorin, glycyrrhizin, Higenamine, ethyl pyruvate, glycyrrhizin, Catalpol, Dihydrotanshinone I, mir-34a, anti-HMGB1 mAb, etc., HMGB1 can reduce inflammation and reduce spinal cord oedema, protect spinal cord neurons and promote functional recovery after SCI by downregulating the expression of HMGB1 and NF-κB (24, 26, 30, 42, 127–129, 131, 146–153). Among these studies, Higenamine induced an increase in M2 macrophages and saw an enhanced the anti-inflammatory effect (147). miR-34a, miR-129-5p, Catalpol and shikonin-induced HMGB1 downregulate the NF-κB signalling pathway involved in the decreased expression of TLR4 (24, 131, 148, 149, 151). Dihydrotanshinone I was shown to protects against SCI *in vivo* through the HMGB1/TLR4/NOX4 pathway. Ethyl pyruvate or glycyrrhizin we shown to reduce spinal cord oedema by reducing the expression of AQP4 in the spinal cord of rats with SCI (129). Furthermore, glycyrrhizin reduced the area of the cystic cavity and glial scar formation (153). However, as mentioned earlier, HMGB1 does not only induce inflammation to aggravate SCI, but it also promotes neuronal regeneration. Transplantation of neural stem cells pre-treated with HMGB1 promotes neuronal survival after SCI in rats. The motor recovery of rats in the HMGB1 combination with neural stem cell group was better than the motor recovery of rats in the single transplantation group and in HMGB1 group (25) (**Table 1**).

**Table 1:** Summary of targeted HMGB1 treatment of SCI.

S.N.	Study Model	Intervention and Dosing Schedule	Observations	References
**1**	SCI model of adult SD rats established by Allen gravity drop method	HBO (8~10L/min, 2.5 ATA, Once or twice a day. Inhaling oxygen for 45 minutes at a time)	HMGB1 mRNA and protein expression water, NF-κB mRNA decreased averagely, and Basso, Beattie and Bresnahan scores increased significantly after HBO intervention.	(146)
**2**	SCI model of contusion in C57BL/6J WT male mice	Higenamine (10 mg/kg, i.p.)	HG decreased the expression of HMGB1, increased the expression of IL-4 and IL-10, promoted the production of HO-1 and the activation of macrophage M2, and increased the BMS score of mice.	(147)
**3**	SCI model of adult SD rats established by Allen gravity drop method	HBO (8~10L/min, 2.5 ATA, Once a day. Inhaling oxygen for 45 minutes at a time)	The production of HMGB1 mRNA, NF-κB mRNA, TLR4 protein and NF-κB mRNA decreased, and BBB score increased. HMGB1 mRNA and TLR4 mRNA were positively correlated with TLR4 protein.	(131)
**4**	SCI models of adult male Sprague-Dawley rats established by gravity drop method	1.5 μ L RAGE antibody (1 μ g/ml) injected into the affected area.	RAGE inhibition reduced the expression of Nestin in MAP-2, a marker of mature neurons, and did not improve the Basso, Beattie, and Bresnahan (BBB) scores after SCI.	(137)
**5**	SCI model of adult male S D rats established by Allen gravity drop method	Shikonin (10-100mg/kg, i.p.)	Shikonin decreased the expression of HMGB1, TLR4 and NF-κB, alleviated inflammatory reaction, spinal cord oedema and increased BBB score after SCI.	(148)
**6**	SCI model of male SD rats established by aneurysm clip compression	Dexmedetomidine intrathecal injection (1, 2, and 4μg/kg)	Dexmedetomidine pretreatment increased the expression of α 7nAChR and acetylcholine and activated PI 3 K/Akt, and increased the BBB score of mice after SCI.	(40)
**7**	SCI model of NOD-SCID female mice established by Infinite Horizon Impactor	anti-HMGB1 (2-16 mg/kg, i.p.)	After treatment with anti-HMGB1 antibody, the destruction of blood spinal screen and the formation of oedema were alleviated, the survival from neurons was increased, and the BMS score was increased, suggesting that the functional recovery was increased. The subsequent hiPSC-NSC transplantation greatly enhanced this recovery.	(127)
**8**	SCI model of adult female S D rats established by Allen gravity drop method	Ethyl pyruvate (50 mg/kg, i.p.) Glycyrrhizin (50 mg/kg, i.p.)	Both EP and GL could effectively inhibit the expression of HMGB1 in spinal cord and the level of serum HMGB1 in SCI rats, inhibit the activation of TLR4/MyD88/NF-κB signal pathway, reduce the overexpression of SCI-related GFAP and AQP4 in spinal cord, improve the motor function of SCI rats, reduce the water content of spinal cord and relieve spinal cord oedema.	(129)
**9**	SCI model of C57 BL/6 female mice established by Infinite Horizon impactor	Catalpol (60 mg/kg, i.g)	Catalpol treatment can reduce the expression of HMGB1/TLR4/NF-κB pathway protein, inhibit apoptosis, reduce inflammation and oxidation, and increase the BBB score of SCI mice.	(24)
**10**	SCI model of adult female S D rats established by Allen gravity drop method	Glycyrrhizin (100 mg/kg, i.p.)	Glycyrrhizin decreased the expression of GFAP, CSPG, HMGB1 and NF-κB in spinal cord, reduced the formation of glial scar and promoted the recovery of hindlimb motor function in rats.	(153)
**11**	SCI model of male SD rats established by modified Tetzlaf lateral spinal cord compression	Glycyrrhizin (100 mg/kg, i.p.) FPS-ZM1 (1 mg/kg, i.p.)	Inhibition of HMGB1 or RAGE effectively reduced the number of harmful proinflammatory macrophages/microglia and increased the number of anti-inflammatory cells after SCI. Inhibition of HMGB1 or RAGE significantly reduced neuronal loss and demyelination after SCI, and improved functional recovery after SCI.	(42)
**12**	SCI model of male SD rats established by aneurysm clip compression	NSCs preconditioned with 1 ng/ml HMGB1 in 3 µl DMEM	NSCs pre-treated with HMGB1-increased the number of functional Nissl bodies in neurons, increased the number of βIII-tubulin^+^ cells in the injured spinal cord of SCI rats, promoted the differentiation of NSCs into neurons and increased BBB score.	(25)
**13**	SCI model of adult SD rats established by Allen gravity drop method	Dihydrotanshinone I (2-4 mg/kg, p.o.)	DI treatment inhibited the levels of TLR4, MyD88, HMGB1, NOX 4, TNF-α, IL-1b, IL-6, iNOS and TOS, increased the level of TAS, alleviated the pathological injury caused by SCI and promoted the recovery of neurological function.	(26)
**14**	SCI model of C57BL/6 female mice prepared by clamp method	Electroacupuncture (1.5 Hz/7.5 Hz,1.0mA) was applied to “jiaji” (EXH-B2) for 10 minutes, once a day for 5-14 days, separately	After electroacupuncture treatment, the expression level of TLR4 protein, HMGB1 and Iba1 protein decreased significantly, the number of Iba1 positive cells and HMGB1/Iba1 copositive cells in spinal cord decreased significantly, and the morphology of microglia in spinal cord changed from overactivated state to resting state. BBB score increased significantly.	(152)
**15**	SCI model of male Wistar rats established by aneurysm clip compression	1 μM ATRA-treated BM-MSCs	ATRA-MSCs increased the levels of Beclin-1 and LC3-II, downregulated the levels of proteins related to HMGB1/NF-κB/NLRP3 pathway, inhibited proinflammatory cytokines, improved the motor activity of hindlimb, and contributed to the survival of neurons, showing a greater beneficial effect than MSCs.	(31)
**16**	SCI model of male SD rats established by aneurysm clip compression	Glycyrrhizic Acid (100 mg/kg, i.p.)	GA significantly decreased the expression of HMGB1 and inflammatory factors after SCI. GA decreased the phosphorylation of p38 and JunN-terminal kinase proteins, but did not decrease their expression levels.	(44)

In summary, HMGB1 plays a multifunctional role in SCI. Inhibition of HMGB1 inhibits nerve inflammation and protects the spinal cord; furthermore, HMGB1 can be used to protect neurons, promote neuronal regeneration and induce neuronal differentiation to promote the recovery of SCI. This information will provide new theoretical support and therapeutic targets for SCI; however, at present, research on treating SCI by targeting HMGB1 is not sufficient, and its specific application scheme still needs to be further explored.

## 3 Discussion

The HMGB1 protein is a highly conserved nonhistone binding nuclear protein. Structurally, HMGB1 is divided into the following three functional regions: the A-box, the B-box and the acidic C-terminus, with similar amino acid repeat sequences and nonspecific DNA-binding sites; the B-box is the functional structural region that causes inflammation, the A-box has a certain antagonistic effect on the B-box, and the C-terminal is involved mainly in regulating the binding affinity of HMGB1 and DNA, mediating gene transcription and chromosome unwinding. In the resting state, HMGB1 is located primarily in the nucleus and it regulates key nuclear activities, including transcription, replication, DNA repair and nucleosome formation, all of which are important for maintaining steady-state cellular function; however, there are no related studies on the structural and functional changes in HMGB1 after SCI.

After SCI, HMGB1 is expressed and released by neurons, microglia/macrophages and ependymal cells. After injury, the expression of HMGB1 is rapidly upregulated and released into the extracellular matrix and circulating blood, where it remains for a long time. Interestingly, in addition to the increased expression of HMGB1 in the acute and subacute phases after SCI, the level of HMGB1 also increased significantly in the chronic phases. The expression of HMGB1 increases after SCI, and it runs through the acute, subacute and chronic stages of SCI, indicating that HMGB1 may affect the severity and recovery process of SCI. In addition, the expression of HMGB1 is still upregulated in the chronic phase after injury, and its specific mechanism and effect still need to be explored. HMGB1 increased in the nucleus and it was then released from the nucleus to the cytoplasm and extracellular matrix; however, the specific time and node of translocation have not been clarified.

In SCI, the secretion and release mechanism of HMGB1 has not been specifically described, but it is generally thought that most HMGB1 comes from the secretion of inflammatory cells and the release of dead neurons. Inflammation can induce many kinds of cells to secrete HMGB1; however, HMGB1 cannot be actively secreted through the conventional endoplasmic reticulum-Golgi secretion pathway utilized by most soluble secretory proteins. Scholars have proposed two mechanisms for secretion. Among these, one mechanism hypothesizes that HMGB1 is packaged into intracellular vesicles, and then HMGB1 is released out of cells after the fusion of vesicles and the cytoplasmic membrane, consistent with the cytoplasmic vacuoles of HMGB1 observed in macrophages after SCI.

After SCI, HMGB1 may promote the active release of inflammatory cells through calcium-mediated signal transduction in inflammation, HSPA1A inhibition of the interaction between HMGB1 and XPO1, MAPK family members, inflammatory bodies, activation of STAT1 and STAT3 regulated by JAK, and upregulated expression of TNF and NF-κB. HMGB1 may also be passively released by necrotic neurons, and the intracellular substances released by necrotic cells can also induce further active secretion of HMGB1.

HMGB1 that is secreted and released into the extracellular space can induce the migration of inflammatory cells, aggravate the inflammatory response by regulating the activation of microglia to the M1 phenotype, and regulate the expression of inflammatory factors through RAGE and TLR2/4. However, the studies of Wang et al. show different results, and the specific mechanism needs to be further explored. In addition, the above studies address the role of HMGB1 only in acute and subacute SCI; however, the expression of HMGB1 is still upregulated in the chronic stage of SCI. Its specific role needs to be further explored, and the possibility that HMGB1 promotes the recovery of SCI cannot be ruled out. Moreover, extracellular HMGB1 can aggravate the inflammatory response after SCI, but the specific changes in nuclear HMGB1 after SCI are not clear, and its specific role remains to be understood.

HMGB1 can aggravate neuronal inflammation and lead to neuronal death, and it is also beneficial to neuronal survival and recovery. However, the specific mechanism by which HMGB1 protects injured neurons and promotes injury recovery is not clear, and possibility that HMGB1 injures neurons indirectly by promoting the inflammatory response, while protecting neurons through direct action has not been excluded. In addition, further studies have found that HMGB1 can promote the growth of neuronal axons and induce the migration and differentiation of neural stem cells. The specific mechanism needs to be further explored. In the chronic stage of SCI, the upregulation of HMGB1 may be involved in regulating neuronal regeneration and promoting motor recovery.

HMGB1 plays a multifunctional role in SCI. Inhibiting HMGB1 inhibits nerve inflammation and protects the spinal cord; furthermore, it can be used to protect neurons, promote neuronal regeneration and induce neuronal differentiation to promote the recovery of SCI. These findings provide a new theoretical basis for studying SCI and new therapeutic targets for SCI treatment. However, at present, research into the treatment of SCI based on targeting HMGB1 is not sufficient, and its specific application scheme still needs to be further defined.

For future research, we suggest focusing on the different roles of HMGB1 in the nucleus and in the extracellular space after SCI, clarifying the specific secretion and release mechanism, and determining the spatiotemporal relationship of HMGB1 expression, the protective effect on neurons, and the mechanism of promoting differentiation.

In summary, HMGB1 plays a complex role in SCI, and its mechanism remains to be understood. Additional research will provide a new theoretical basis and therapeutic target for the treatment of SCI.

## Author contributions

Conceptualization, data curation, writing-original draft preparation, writing-review and editing: all authors; supervision: KC. Funding acquisition: KC. All authors contributed to the article and approved the submitted version.
